# A Newly Designed Automatically Controlled, Sterilizable Flat Panel Photobioreactor for Axenic Algae Culture

**DOI:** 10.3389/fbioe.2021.697354

**Published:** 2021-07-01

**Authors:** Tobias Fuchs, Nathanael D. Arnold, Daniel Garbe, Simon Deimel, Jan Lorenzen, Mahmoud Masri, Norbert Mehlmer, Dirk Weuster-Botz, Thomas B. Brück

**Affiliations:** ^1^Werner Siemens-Chair of Synthetic Biotechnology, Technical University of Munich, Garching, Germany; ^2^TUM-AlgaeTec Center, Technical University of Munich, Taufkirchen, Germany; ^3^Bürkert Werke GmbH & Co., KG, Systemhaus Ingelfingen, Ingelfingen, Germany; ^4^Institute of Biochemical Engineering, Technical University of Munich, Garching, Germany

**Keywords:** *Chlorella sorokiniana*, flat panel gas-lift, microalgae, photobioreactor, sterilizability

## Abstract

In context of the global climate change, microalgae processes are gaining momentum as a biotechnological tool for direct fixation and valorization of greenhouse gases. Algae have the metabolic capacity to photosynthetically convert CO_2_ into high value products, such as food additives, under economic boundary conditions. High cost, commercial flat panel gas-lift bioreactors for microalgae cultivation at laboratory scale provide either small volumes or no sterile operation, which limits academic research. This brief report presents initial data for a new type of sterile operating flat panel gas-lift bioreactor with a unique asymmetrical U-shape. It utilizes automatable process control technologies that adhere to industrial standards to enhance data reproducibility and aid industrial scale up. The practicability was demonstrated using a *Chlorella sorokiniana* cultivation, which showed the typical growth behavior. Due to the sophisticated implemented control engineering technology, pivotal parameters as pH and temperature can be determined within a range of ±0.1 units, which was confirmed experimentally. The new flat panel gas-lift photobioreactor presented in this brief report fills the technology gap at laboratory scale with an autoclavable volume of 7.2 L. Moreover, it is easy to rebuild by means of the hereby provided blueprint, while exhibiting a six-fold cost reduction compared to commercially available flat panel photobioreactors.

## Introduction

Excessive anthropogenic CO_2_ emissions are the main cause of the greenhouse gas effect that results in progressive global warming. It represents a focal challenge for mankind to preserve our planets ecosystem. To alleviate climate change effects there is a demand for innovative technologies that can actively fix and reduce atmospheric CO_2_ (Goli et al., 2016; Vo Hoang Nhat et al., 2018). Microalgae are a taxonomically diverse group of photosynthetic microorganisms which, due to their superior photosynthetic efficiency, can convert CO_2_ up to ten times faster into biomass than any vascular, terrestrial plant (Goli et al., 2016; Adeniyi et al., 2018). As microalgae can be cultivated on marginal lands using waste- or salt water, their mass cultivation does not impact agricultural activity or generate land use change associated with negative impacts on biodiversity. Since microalgae biomass contains value adding products, these whole cell biocatalysts represent a yet underexploited toolbox for the efficient CO_2_ fixation and value creation under economic and ecologic boundary conditions. Depending on the selected algae strain and its respective cultivations conditions, microalgae biomass contains varying concentrations of multiple value adding products such as sugars, lipids, proteins, pigments, vitamins, or extracellular polymers (Chisti, 2007; Griesbeck and Kirchmayr, 2012; Woortman et al., 2020a). Examples for microalgae biomass valorization encompass diverse applications, such as high folate (vitamin B9) concentrations accumulating in the rapidly growing green algae *Picochlorum* sp. but also aviation biofuel production using oleaginous microalgae like *Microchloropsis salina* (Chew et al., 2017; Woortman et al., 2020a, b).

These aforementioned oil producing microorganisms can accumulate up to 60% of their dry biomass as lipids with substantially higher oil yields per hectar than currently favored feedstock (Chisti, 2007). Therefore, oleaginous microalgae may help to counteract the second central challenge of humanity as well, which is to meet the rising energy demands coupled with diminishing fossil fuel supplies (UN Energy Statistics, 2018). Extracted oil from algae biomass can serve as raw material for the production of biodiesel through transesterification. During the conversion process, triacylglycerides are separated to fatty acid methyl esters and glycerol under the presence of a short chain alcohol, most commonly methanol, and a catalyst. Subsequently, the oil free biomass waste stream can be processed into further energy carriers like biobutanol by means of fermentation with *Clostridia* sp. bacteria or biogas through gasification or pyrolization (Potts et al., 2012).

In order to use microalgae cultivation as a technology platform, however, entails identification of suitable microalgae strains and their selection for a respective application, which subsequently has to be integrated into an industrial process by iterative scale up in standardized open- or closed system photobioreactors. While open pond cultivation approaches allow for scale up and production, disadvantages involve contamination and low control over experimental parameters. Enclosed systems on the other hand offer lower cultivation volumes but higher levels of control over experimental conditions instead, which renders them more suitable for process development (Chisti, 2007). Various designs of closed photobioreactors have been established: tubular (Molina et al., 2001; Hulatt and Thomas, 2011), flat panel (Hu et al., 1998; Lakaniemi et al., 2012), column (Béchet et al., 2013), and biofilm (Blanken et al., 2014) systems. Flat-panel configurations are among the best designs due to a high surface area to volume ratio in addition to a short light path length while consuming less energy than tubular systems (Zou et al., 2000; Jorquera et al., 2010).

The initial processes of algae identification and laboratory process characterization are commonly conducted by academic groups, which due to limited funds often use different self-constructed cultivation vessels and conditions, which conventionally do not allow sterile operation with medium to high vessel volumes of >5 L. Axenic cultivation is of great importance to eliminate external influences and contaminations, potentially allowing for the development of Food and Drugs Administration agency regulation compliant medical and food products (Chew et al., 2017). Furthermore, sterile operation conditions are vital for research when examining co-cultivation of specific microorganisms or single strain experiments (Yong et al., 2021). Examination of the literature data used for composing Table 1 indicates, that with increasing culture volume photobioreactor assemblies either are not sterilizable or require extensive technical effort to provide a sterile operation mode. Additionally, custom-developed closed photobioreactors documented in literature are mostly square shaped, potentially promoting the emergence of disadvantageous dead zones, where the medium admixture is flawed (Sierra et al., 2008; Tamburic et al., 2011; Gifuni et al., 2018). In that regard, our photobioreactor concept fills a technology and volume gap, as it can be disassembled easily and each component is sterilizable by itself as well as the entire system. Moreover, the sterilization process is using standard equipment such as a laboratory autoclave.

**TABLE 1:** Comparison of the photobioreactor presented in this brief report with selected flat panel photobioreactors in the laboratory scale as described in literature regarding their volume and sterilizability.

**Volume [L]**	**Sterilizable**	**Reactor form**	**References**
0.3	+*	Erected flat rectangle	Gifuni et al., 2018
0.4	+	Square with rounded edges	Hulatt et al., 2017
0.9	+	Square with inner comb shape	Wang et al., 2019
>1	N.D.	V-shaped converging bottom	Jiang et al., 2020
3.0	+	Square with rounded edges	Nyberg et al., 2015
3.4	−	Rectangle shaped	Barbosa et al., 2005
7.0	−	−	Lakaniemi et al., 2012
7.0	−	Stacked floors	Cheah et al., 2020
**7.2**	+	**Square with asymmetrical U-shape**	**This brief report**
15.5	−	Rectangle shaped	Yang et al., 2014
18.5	−	Cube shaped	Choi et al., 2013
30.0	−	Square shaped	Singh Khichi et al., 2018
48.0	+**	Stacked pipes	Li et al., 2015
86.0	−	Rectangle shaped	Mhatre et al., 2018

(*) chemical sterilization, (**) ozone sterilization.

Conversely, commercially available photobioreactors at laboratory scale with the desired operation features are cost intensive and are only available in small volume capacities of less than 4 L^12,3^. A summative comparison of the new photobioreactor’s specifications presented in this brief report with established flat panel bioreactors systems operated as gas-lift reactors can be found in Table 2.

**TABLE 2:** Comparison of the photobioreactor presented in this brief report with selected commercially available photobioreactors operated as gas-lift reactors in the laboratory scale.

**Feature**	**Photobioreactor of this report**	**Subitec GmbH^4^**	**Infors GmbH^1^**	**Biostream International BV^3^**	**PSI spol. s r.o.^2^**
Prevention of dead zones	**+**	**+**	**+**	**+**	**+**
Autoclavability	**+**	**−**	**+**	**+**	**+**
Working volume > 7L	**+**	**−**	**−**	**−**	**−**
Cost-effective <10,000 €	**+**	**+**	**−**	**−**	**−**

*^1^Labfors 5 Phototrophic 1.9 L TV, Infors GmbH, Sulzemoos, Germany. ^2^Photobioreactors FMT 150, PSI spol. s r.o., Drásov, Czechia. ^3^Biostream International BV, Doetinchem, Netherlands. ^4^Flat Panel Airlift Photobioreaktor, Subitec GmbH, Stuttgart, Germany.*

To circumvent issues of current commercial systems, a new design of flat panel gas-lift photobioreactor was developed in this brief report. Its unique asymmetrical U-shape prevents formation of dead zones, which are induced by microalgae biomass precipitation at high cell densities commonly observed in the late exponential growth phase. Supplementary Figure 1 serves to illustrate, how the unique geometry of the new flat panel air lift photobioreactor allows for enhanced mixing of the cultivation medium and distinguishes itself from commercially available equivalents.

In addition, the new flat panel photobioreactor vessel can be sterilized in a conventional laboratory autoclave, therefore enabling axenic microalgae cultivation, allowing for algae process optimization targeted at the food- and pharmaceutical sector. Due to its straightforward architecture, this 7.2 L photobioreactor is easily constructed and exhibits a six-fold cost reduction compared to commercially available systems. Owing to the implemented automated, industrially standardized systems control technology, it is not only possible to manipulate different growth parameters, such as temperature, pH value and light intensity, but also to control light and temperature cycles, thereby enabling complex climate simulations, which are key for iterative process scaling toward outdoor cultivation deployment at industrial scales.

## Materials and Methods

### Photobioreactor Design

The photobioreactor was designed and constructed making use of the CAD-software Autodesk (2019.02, Autodesk Inc., CA, United States).

For simplified sterilization purposes, the size of the photobioreactor (see Supplementary Figures 2, 3) was set to fit into a conventional laboratory autoclave. Furthermore, the metal frame includes a closed tubing system which permits the application of a liquid based cooling medium. One 4-mm-thick safety glass pane on each side serve as the outer boundary for the flat panel photobioreactor with a distance of 40 mm. The latter are pressed onto the metal rod by clamping jaws after a 1 mm thick silicone seal has been attached. Additionally, an external cooling circuit was implemented, which is completely isolated from the cultivation medium. Advantages of this reactor architecture include the potential utilization of hazardous coolants, which provide a higher cooling performance than water. As a result, the cultivation of cryophilic Snow and Ice Algae, which are of interest for carotenoid pigment production for the food industry is enabled with this panel bioreactor.

**FIGURE 1:**
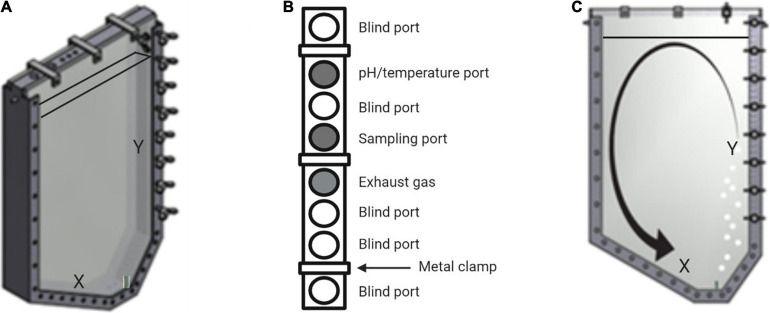
Schematic view of the novel flat panel gas-lift photobioreactor. **(A)** Frontal view of the photobioreactor. X indicates the sloping edge, while Y illustrates the area of maximum buoyancy above the sparger. These two characteristics are responsible for adequate flow formation and prevention of dead zones. The fill level line indicates a volume of 7.2 L. **(B)** Top view of the eight inlet ports of the reactor. Three metal clamps seal the upper part of the device. For this study, only the three shaded inlets were used for pH- and temperature measurements, sampling and for gas exhaust. **(C)** Schematic medium flow within the bioreactor. The gas input via the sparger results in a buoyancy at Y. The sloping edge X pushes the liquid stream toward the sparger, resulting in a circular confection that inhibits sedimenting of biomass.

As indicated in Figure 1, the gas supply as well as the harvest valve are located on the right hand side at the lowest point of the photobioreactor. Resulting from the asymmetrical U-shape paired with the lateral positioning of the air supply, the mixing is improved in contrast to other square shaped reactors (Sierra et al., 2008; Tamburic et al., 2011; Gifuni et al., 2018). Due to the sloping edge X (see Figure 1), the liquid flow created by the buoyancy is redirected toward the sparger. The gas flow is in turn deflected toward the Y edge. Therefore, sedimentation of biomass and formation of dead zones can be prevented. At the top of the reactor, eight inlet ports (M20 × 1,5) allow the assembly of sampling ports as well as different sensors, e.g., for pH and temperature measurements.

The illumination on both sides was embedded into the outer cladding. For this purpose, the two LED Mini-Matrices were mounted on a four mm thick Plexiglas plate and screwed to the outer cladding. The luminous flux output of the LED MiniMatrix can be varied between 0–750 μmol m^–2^ s^–1^, directly adjusted by the engineering control system or controlled individually.

The software tool type 8922 (Christian Bürkert GmbH & Co. KG, Ingelfingen, Germany) was utilized to control pH, temperature, gas mixture, and gas flow rate.

The following hardware from Christian Bürkert GmbH & Co. KG was installed: MFC type 8742-15.0 NL/min – air, MFC type 8742-1.0 NL/min – CO2, motor valve type 3285 – positioner – DN10. An ME43 – Feldbus-Gateway (Christian Bürkert GmbH & Co. KG, Ingelfingen, Germany) and Type ME2X – System Control Unit (Christian Bürkert GmbH & Co. KG, Ingelfingen, Germany) was used as an interface between the measured data and the resulting circuit of the valves according to the manufacturers’ instructions. An autoclavable POLILYTE PLUS H ARC 325 sensor (Hamilton Germany GmbH, Gräfelfing, Germany) was used for pH and temperature measurement inside the photobioreactor. The lighting was implemented by two LED MiniMatrices (Spectral color of 6500K, max. 750 μmol m^–2^ s^–1^, 504 LEDs, 27 × 42 cm, 24V; LUMITRONIX^®^ LED-Technik GmbH, Hechingen, Germany).

### Sterility Test

To test for autoclavability, the photobioreactor was filled with 7.2 L of yeast nitrogen base (YNB) medium containing the following components: 1.7 g/L YNB, 5 g/L ammonium sulfate, and 10 g/L glucose. The agar plates were prepared with yeast extract peptone dextrose (YPD) medium containing the following components: 1% yeast extract, 2% peptone, 2% glucose, and 2% agar (all chemicals provided by Carl Roth GmbH + Co. KG, Karlsruhe, Germany).

The fully assembled liquid media containing reactor was sterilized in an autoclave (Systec VE-150, Systec GmbH, Linden, Germany) for 20 min at 120∘C and used for algae cultivation afterward for 7 days. Finally, samples were taken and streaked out on agar plates to demonstrate sterility.

### Temperature Control

The temperature was regulated by a water based circuit using a cooling thermostat/cryostat regulator (UWK 300, VACUUBRAND GmbH & Co KG, Germany) connected to the photobioreactor’s in- and outlets. Liquid flow of the coolant was 0.08 L/s regulated by the control units of Bürkert.

### Reactor Mixing

In order to evaluate the mixing, the time was stopped 5 times until 100 mg/100 ml of a red food dye (Ponceau 4R, funfood4you, Germany) was completely dissolved in the water filled reactor.

### Cultivation of *Chlorella sorokiniana*

Throughout our study we used an axenically purified strain of *Chlorella sorokiniana* ATCC 22521 (Thomas and Syrett, 1976). The absence of microbial contaminants and the axenic nature of the strain was controlled on solid agar based stock cultures by microscopic examination (Zeiss AxioLab, Carl Zeiss AG, Oberkochen, Germany) as well as flow cytometry (Biorad S3 Sorter, Bio-Rad Laboratories, Inc., Hercules, United States) on a weekly basis. *C. sorokiniana* was cultivated in Bold Basal Medium (BBM). The BBM was produced as published, but without organic nutrients, vitamins, or complex components (Bischoff, 1963). After sterilization, the 7.2 L medium of the photobioreactor was inoculated with a preculture, resulting in an optical density of 0.1 at 750 nm. During the following 7 days, the algae were cultivated at a pH of 7.2, 200 μmol m^–2^ s^–1^, 0.133 vvm of gas input and a temperature of 26∘C. The OD_750_ was measured daily. The standard deviation of the biological triplicates was calculated with the excel command “stabw.”

## Results

### Sterilization

In biotechnological industry processes, axenic single strain cultures are preferred in order to enhance process control. Hence, the validation of sterile operations is critical to enable algae process design and optimization for the food and pharma industry. Therefore, the photobioreactor in this brief report was designed in a manner that it fits into a conventional laboratory autoclave. The following experiment should verify said sterile operation. In contrast to commonly utilized algae cultivation media, which conventionally contain inorganic C- and N-sources, the sterility was tested under adverse heterotrophic conditions, using glucose containing YNB media. Sterile samples (syringe) were taken from the photobioreactor operated at 26∘C with sterile air sparging (0.133 vvm) 7 days after an autoclaving cycle (20 min at 121∘C) and were subsequently streaked out onto a YPD media agar plate. Supplementary Figure 4 depicts the inoculated plate after an incubation period of 3 days at 28∘C. As illustrated, no microbial colonies could be identified on the inoculated plate during this period, confirming, that the novel photobioreactor provides complete sterility and aptitude for the cultivation of axenic algae cultures.

### Temperature Control

The exhaust heat radiating from the LED based illumination units guarantee a constant heat input into the reactor system. To counteract excessive heat intake, controlled cooling is crucial to ensure stable growth conditions. Therefore, a regulated thermostat/cryostat is employed, which pumps cooling water through a main cooling circuit according to the current temperature.

In order to assess the cooling capacity of the system, the time frame for the photobioreactor required to cool the filling volume of 7.2 L from 22∘C to 21∘C was measured. The heat exchange surface of the frame between the coolant (11∘C) and the filling media is 63 cm^2^. While the system of a standard laboratory scale stirred-tank bioreactor (Labfors 5, Infors GmbH, Sulzemoos, Germany) used for comparison needs 2:30 min to cool 1 L by 1∘C, the reactor introduced in this brief report records a time of 3 min for the entire filling volume of 7.2 L.

In order to compare and reproduce microalgae growth experiments, a constant temperature profile throughout the cultivation process is beneficial. Hence, undesirable stress conditions triggering respective adaptations can be minimized, which might negatively impact cell growth. The exhaust heat radiating from the LED based illumination coupled with the room temperature represented the heat input into the reactor system. To demonstrate that the reactor is capable of complying with requirement, it should maintain the target temperature of 25∘C as accurately as possible. Therefore, the heat regulation of the novel LED-illuminated (200 μmol m^–2^ s^–1^) photobioreactor system was monitored over the course of 12 h with water, only. Strikingly, the temperature varied merely ±0.1∘C from the specified target value (Supplementary Figure 5).

### Gassing and pH Control

**FIGURE 2:**
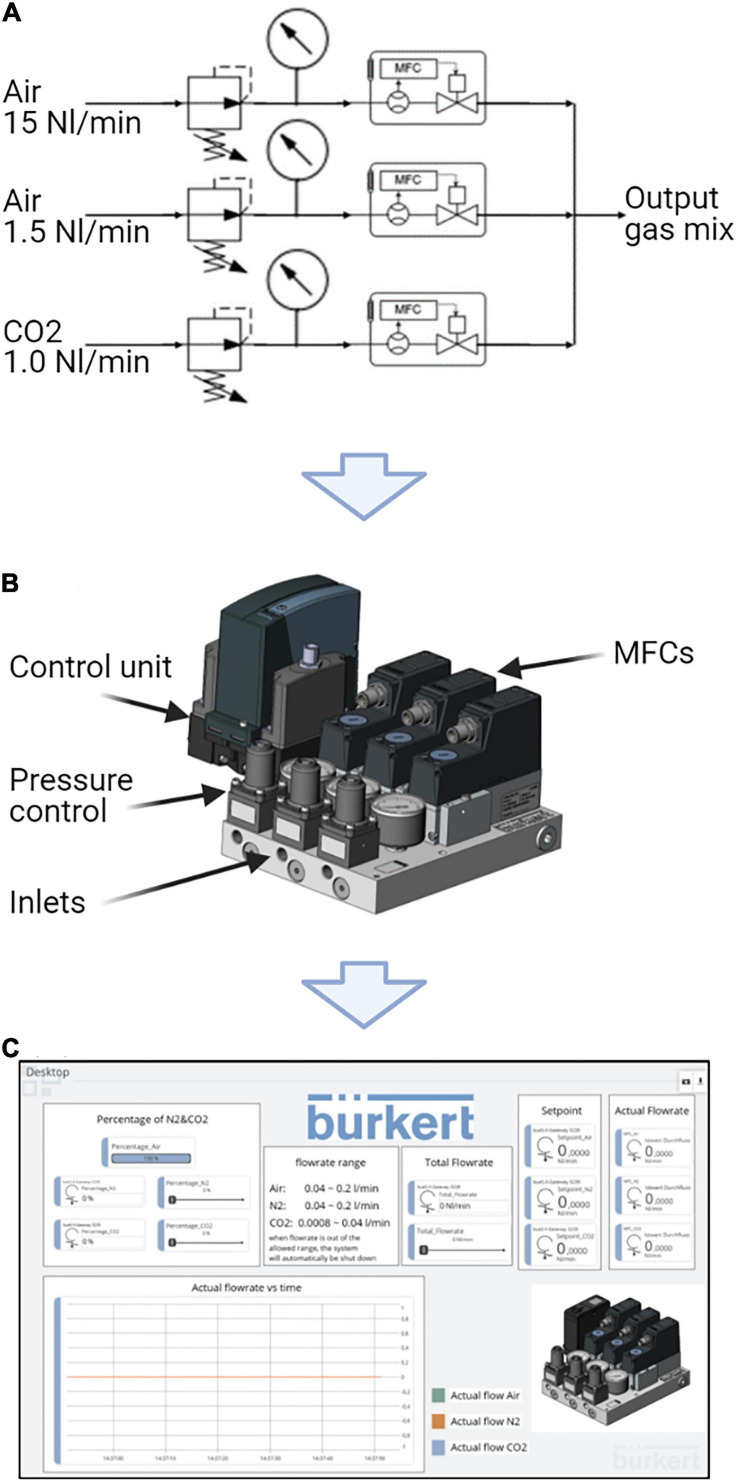
Gassing unit of the novel flat panel gas-lift photobioreactor. **(A)** Schematic structure of the fumigation unit, including three parallel mass flow controllers (MFCs). **(B)** Outer appearance of the fumigation unit developed in cooperation with the cooperation partner Christian Bürkert GmbH & Co. KG. **(C)** Overview of an exemplary user interface, which can be adjusted according to the experimental requirements. Features of the software comprise real time parameter monitoring and creation of quick access buttons.

In order to provide adequate gassing of the novel photobioreactor, a customized gas mixing system was developed in accord with industrial standards in cooperation with the company Christian Bürkert GmbH & Co. KG. Figure 2 illustrates the gas mixing system, consisting of a compact block on which three mass flow controllers (MFC) and pressure regulators are installed in parallel. At the inlet, compressed air (fed with three bar), oxygen (not connected), and carbon dioxide (fed with one bar) into the respective MFC. The MFCs are controlled with the support of the Bürkert software tool. Mixture of the respective gasses takes place in the joint outlet, where they are subsequently led to the photobioreactor through the supply line, which is attached on the bottom of the reactor.

During the microalgae cultivation, the supply of compressed air is kept at a constant level of 0.133 vvm ensuring an optimal circulation of the microalgae culture. Photosynthetically active microalgae consume dissolved CO_2_ during growth, causing an increase in the pH. Based on this interaction, the pH is mediated by CO_2_ pulses in the order of 0.028 vvm to the medium. During cultivation, the software detects when the threshold is exceeded and mixes CO_2_ into the feed stream by opening the CO_2_-MFC, which causes the pH value to drop again. To determine the accuracy of this controlling entity, the pH value was monitored during cultivation. The data displayed a fluctuation of ±0.05 around the set point of pH 7.2 (Supplementary Figure 6).

### Reactor Mixing Characteristics

To investigate the influence of the asymmetrical U-shape on the mixing, a red food dye was poured into the water containing reactor and the time was stopped until complete homogenization was achieved. Hereby the average mixing time was determined as 34,47 ± 7,37 s. The Supplementary Video 1 provided in the supplements serves to illustrate the hydraulic characteristics of the red dye inside the reactor.

### Cultivation of *Chlorella sorokiniana*

Functionality of the new photobioreactor could be demonstrated by cultivating the literature established eukaryotic green algae *C. sorokiniana* ATCC 22521 (Thomas and Syrett, 1976). This specific algae strain was selected due to its rapid growth and biomass formation. Growth was recorded at constant incident irradiation of 200 μmol m^–2^ s^–1^ (pH 7.2, 26∘C, 0.133 vvm, layer thickness of 3 cm) over a period of 7 days until the culture reached the stationary phase (see Supplementary Figure 7). The growth curve was plotted, exhibiting typical lag-, exponential-, and stationary phases. Additionally, the maximum increase in optical density (OD_750_) was determined. Inherent to this algae strain, growth could be observed until the 4th day of cultivation. The maximal increase rate of 2.68 (OD_750_) was reached between day two and three. Throughout the entire cultivation period, no dead zone or biofilm formation on the illuminated glass plates could be detected, hence emphasizing the benefits of the asymmetrical U-shape design.

## Discussion

The newly designed, autoclavable flat panel gas-lift photobioreactor presented in this brief report offers a low-cost alternative for laboratory applications at the 7.2 L scale. Moreover, the presented photobioreactor utilizes a set of industrially standardized and automatable process control technologies, which will enhance process development and data reproducibility as well as integrity, which provides for accelerated iterative scale up procedures toward industrial deployment. In order to accelerate iterative scale up of microalgae processes for academic research, all of the necessary data to rebuild this photobioreactor was deliberately fully disclosed in this brief report. The unique asymmetrical U-shape design provides enhanced mixing of the cultivation medium, which prevents formation of dead zones at high cell density biomass production scenarios required for an economically viable technology platform. Additionally, the sophisticated control engineering of this bioreactor offers an alternative open platform for microalgae cultivation at the intermediate laboratory scale of 7.2 L.

In this flat panel based photobioreactor concept, illumination is performed from both sides, providing a maximum of light energy intake for efficient production of biomass and intracellular products, such as lipids or carotenoid pigments. Due to its modular construction, illumination from one individual side is feasible, allowing the determination of light dependent growth parameters, which are critical for iterative process and reactor scale up (Pfaffinger et al., 2016, 2019; Koller et al., 2017). The former is only applicable in early growth phases, since a relatively high glass panel thickness of 40 mm was chosen.

Functionality of the novel photobioreactor was tested and verified by performing batch cultivation experiments with *C. sorokiniana*, a rapidly growing microalgae that exhibited conventional microbial growth stages in these experiments. In addition, an accuracy of temperature and pH control comparable to other reactors was demonstrated. Through utilization of glucose containing medium, highest standards where applied during the sterilization assay. The sterility of the reactor enables the axenic cultivation of single algae strains including recombinant microalgae, rendering it appropriate for research and development in the field of food and pharmacology. Further sophistication of this photobioreactor system could involve incorporation of circadian light cycles and temperature shifts or the implementation of exhaust gas measurements for industrial relevant process development.

## Data Availability Statement

The original contributions presented in the study are included in the article/Supplementary Material, further inquiries can be directed to the corresponding author/s.

## Author Contributions

TF, NM, and TB conceived the experiment and analyzed the data. TF wrote the manuscript. TF conducted the experiments and was supported by NA, SD, and DG. JL, NA, NM, and MM reviewed writing. TB and DW-B supervised the project and reviewed the manuscript. All authors contributed to the article and approved the submitted version.

## Conflict of Interest

The authors declare that this study received funding from Christian Bürkert GmbH & Co. KG. The funder was not involved in the study design, collection, analysis, interpretation of data, the writing of this article, or the decision to submit it for publication. SD was employed by the company Christian Bürkert GmbH & Co. KG. The remaining authors declare that the research was conducted in the absence of any commercial or financial relationships that could be construed as a potential conflict of interest.
