# Supplementary material for: A Newly Designed Automatically Controlled, Sterilizable Flat Panel Photobioreactor for Axenic Algae Culture
Source: Front Bioeng Biotechnol. 2021 Jul 1;9:697354. doi: 10.3389/fbioe.2021.697354 (PMC8280782; doi:10.3389/fbioe.2021.697354)
Supplement: Supplementary file 2 [file Data_Sheet_1.docx]

Supplementary Material


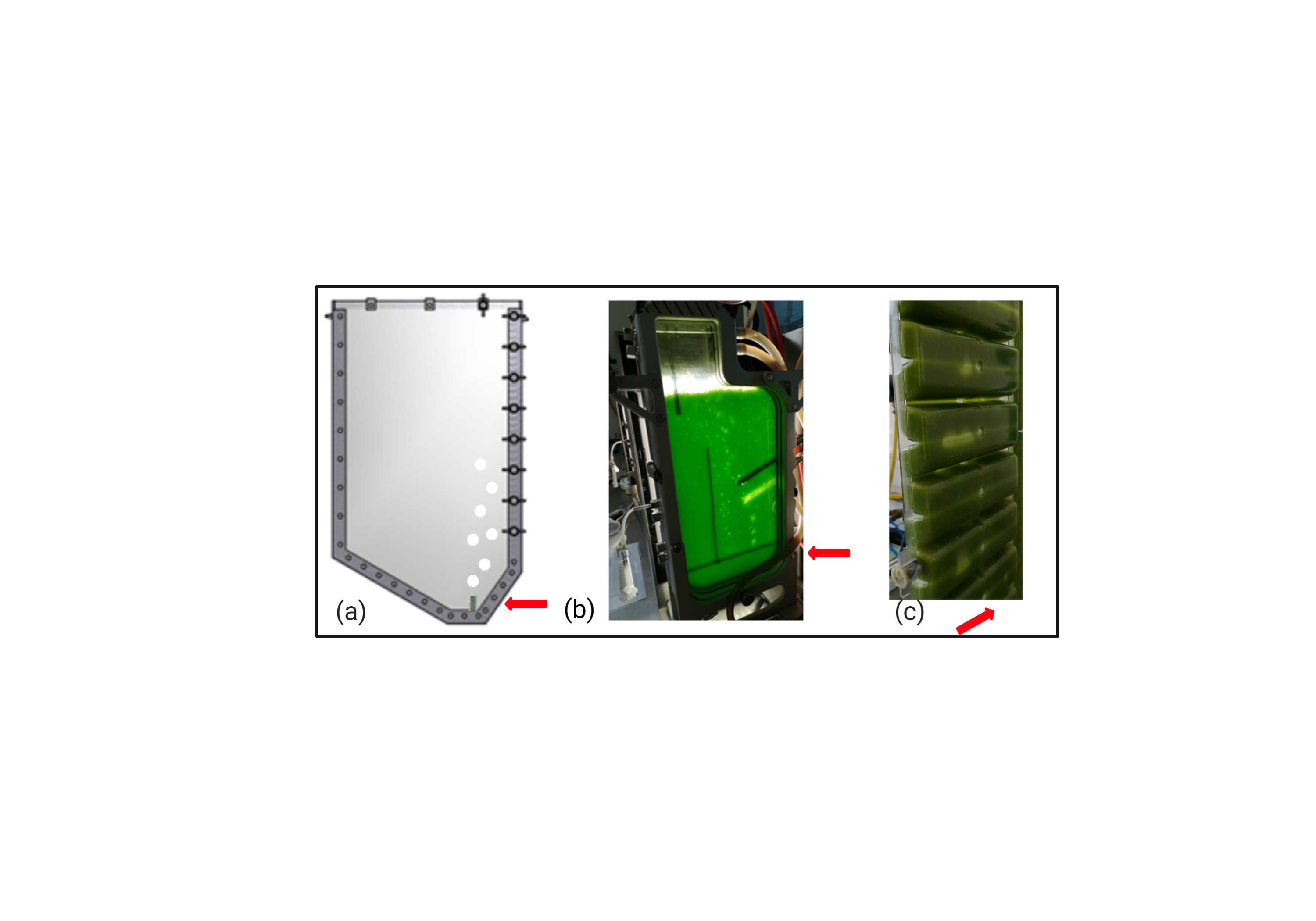


**Figure S1.** Schematic design concepts of the new flat panel photobioreactor in comparison to selected commercially available devices. Gas input is indicated by the red arrows. (a) Schematic architecture of the novel flat panel photobioreactor with its unique asymmetrical U-shape and sloping egde features. (b) Schematic architecture of the similar Infors GmbH1 and Biostream International BV^3^ systems, as presented on their commercial online platforms. (c) Schematic architecture of the Subitec GmbH flat panel gas-lift photobioreactor^4^.

**
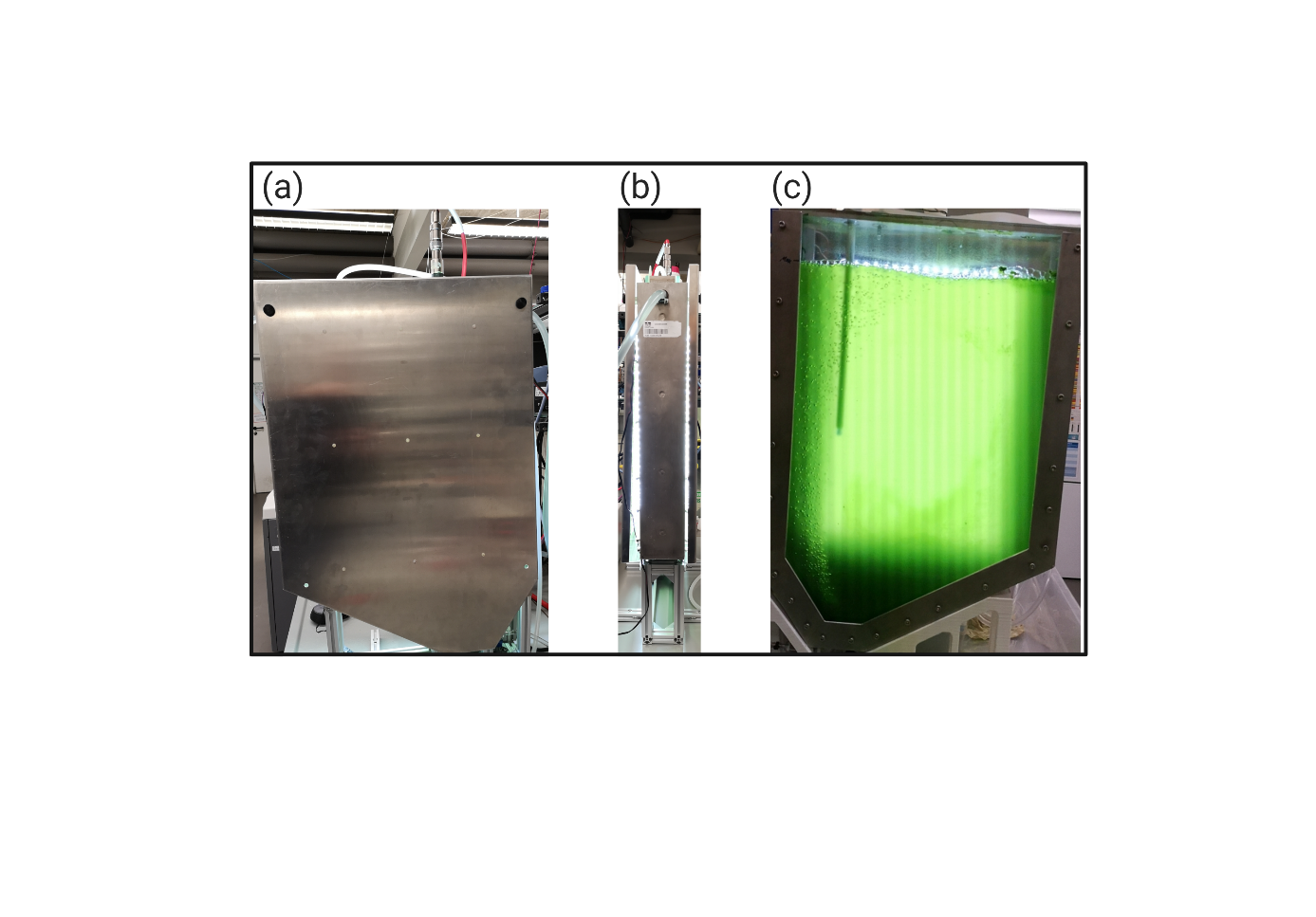
**

**Figure S2.** Architecture of the novel flat panel gas-lift photobioreactor. (a) Front view of the closed photobioreactor with attached metal cover containing the LED light panels. (b) Lateral view of the novel device with attached metal light shielding. (c) Front view of the photobioreactor without metal light shielding.

**
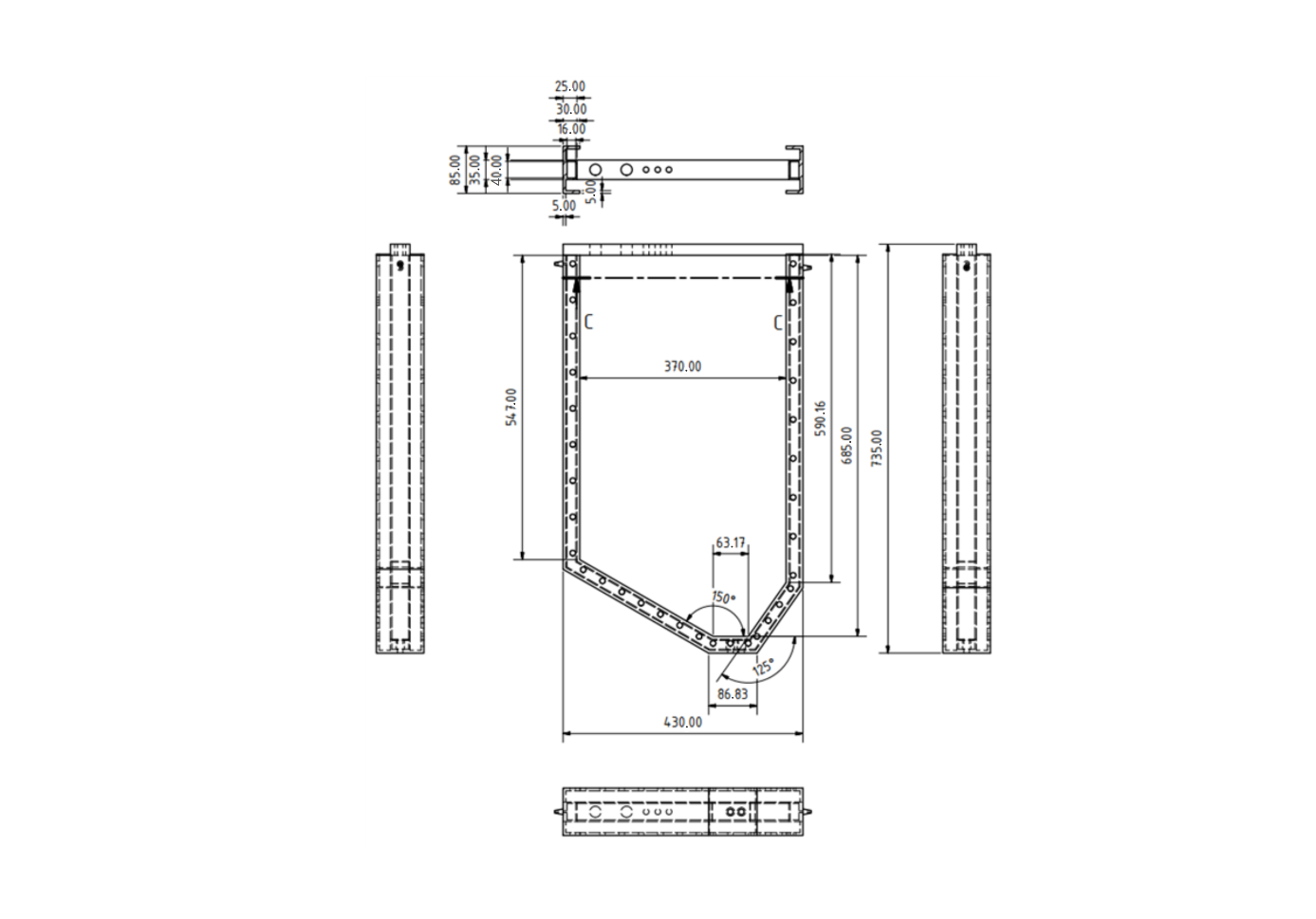
**

**Figure S3.** Blueprint of the novel flat panel photobioreactor. All the necessary measurements [mm] to rebuild the novel flat panel photobioreactor in this study can be derived from this figure.

**
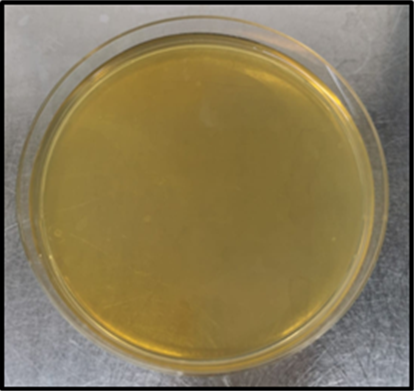
**

**Figure S4.** Sterility test of the novel flat panel photobioreactor. The bioreactor was autoclaved with YNB medium and incubated at 26 °C with 0.133 vvm sterile air sparging for seven days, then samples were drawn and streaked out onto a YPD-agar plate. After an incubation period of three days at 28°C, no colonies were detectable.


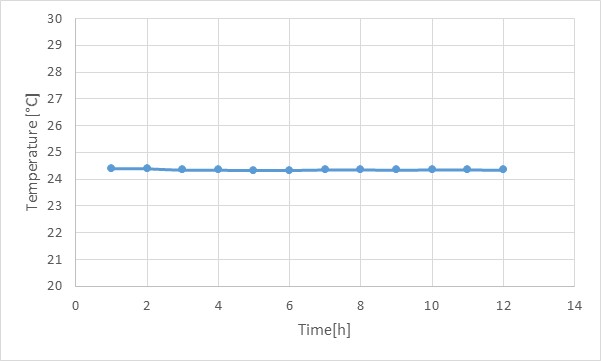


**Figure S5.** The measured temperature curve over 12 hours is indicated.


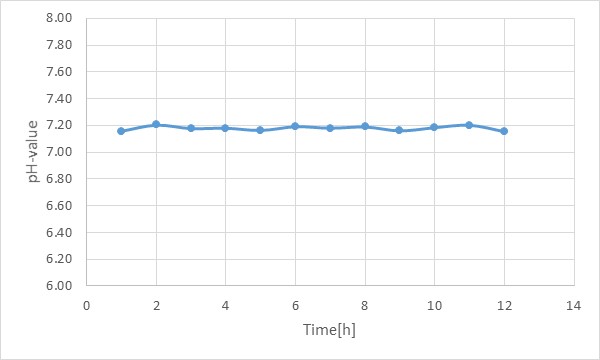


**Figure S6**. The measured pH value curve over 12 hours is indicated.

**
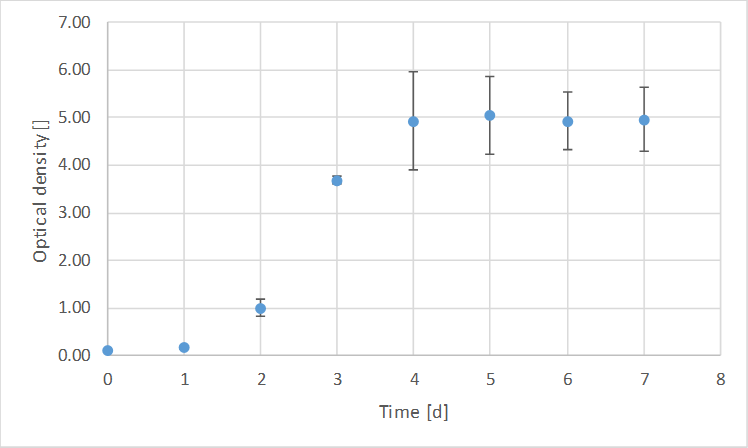
**

**Figure S7.** Growth curve of the microalgae Chlorella sorokiniana ATCC 22521 in the novel flat panel gas-lift photobioreactor. Algae cells have been inoculated with an initial starting OD750 of 0.1 and cultivated for seven days at 26 °C in Bold Basal Medium (BBM) with 200 µmol m-2 s-1 (pH 7.2, 26 °C, 0.133 vvm). Samples have been taken every 24 h. The growth curve exhibits typical lag-, exponential growth- and stationary phases.
